# Supplementary material for: Identification of photoperiod-induced specific miRNAs in the adrenal glands of Sunite sheep (Ovis aries)
Source: Front Vet Sci. 2022 Jul 22;9:888207. doi: 10.3389/fvets.2022.888207 (PMC9354845; doi:10.3389/fvets.2022.888207)
Supplement: Supplementary file 1 [file Data_Sheet_1.ZIP › Supplementary file/miRDeep2 file.zip!/miRDeep2 file/Know_Structure/SP42A1/oar-mir-16b.pdf]

miRBase precursor : oar-mir-16b
Total read count : 31013
oar-miR-16b read count : 30965
remaining reads : 48

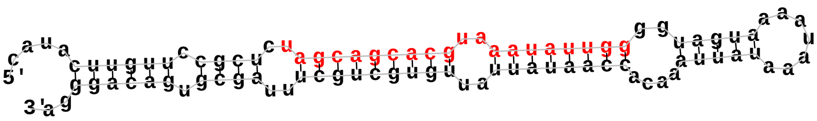

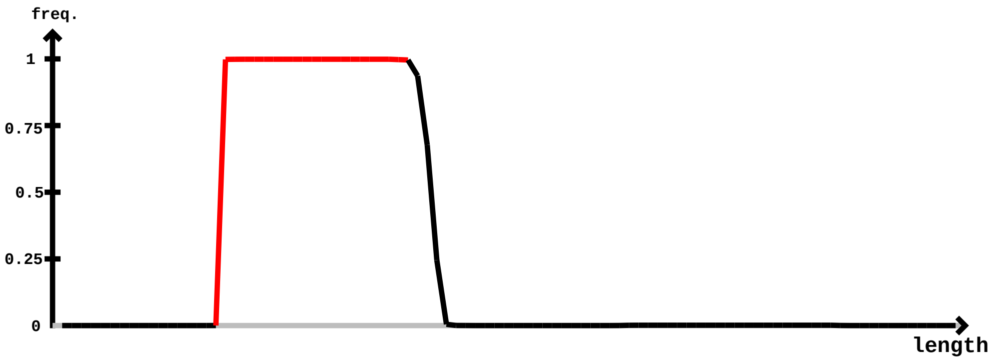


```
                                oar-miR-16b
5'- cauacuuguuccgcucuagcagcacguaaauauugggguaguaaaauaaauauuaaacaccaauauuauugugcugcuuuagcgugacaggga   -3'   exp
    ....((((((.((((..(((((((((..((((((((..(((((.......)))))....))))))))..)))))))))..)))).))))))..   reads   mm   sample
    ...............cuagcagcacguaaauauugg.........................................................   2       0    seq
    ...............cuagcagcacguaaauauugggg.......................................................   2       0    seq
    ...............cuagcagcacguaaauauuggggu......................................................   3       0    seq
    ................uagcagcacguaaauauu...........................................................   43      0    seq
    ................uagcagcacguaaauauug..........................................................   47      0    seq
    ................uagcagcacguaaauauugg.........................................................   1840    0    seq
    ................uagcagcacguaaauauuggA........................................................   7507    1    seq
    ................uagcagcacguaaauauuggg........................................................   530     0    seq
    ................uagcagcacguaaauauuggAg.......................................................   11866   1    seq
    ................uagcagcacguaaauauugggg.......................................................   1518    0    seq
    ................uagcagcacguaaauauuggggu......................................................   2961    0    seq
    ................uagcagcacguaaauauuggAgu......................................................   4485    1    seq
    ................uagcagcacguaaauauuggggua.....................................................   137     0    seq
    ................uagcagcacguaaauauugggguag....................................................   4       0    seq
    ................uagcagcacguaaauauugggguagu...................................................   6       0    seq
    ................uagcagcacguaaauauugggguagua..................................................   1       0    seq
    .................agcagcacguaaauauuggA........................................................   2       1    seq
    .................agcagcacguaaauauuggAg.......................................................   10      1    seq
    .................agcagcacguaaauauuggAgu......................................................   2       1    seq
    .................agcagcacguaaauauuggggu......................................................   1       0    seq
    ..................gcagcacguaaauauuggAg.......................................................   2       1    seq
    ..................gcagcacguaaauauugggg.......................................................   1       0    seq
    ..................gcagcacguaaauauuggAgu......................................................   2       1    seq
    .........................................................caccaauauuauugugcugcuu..............   1       0    seq
    .........................................................caccaauauuauugugcugcuuu.............   6       0    seq
    ..........................................................accaauauuauugugcugcuu..............   1       0    seq
    ..........................................................accaauauuauugugcugcuuu.............   20      0    seq
    ..........................................................accaauauuauugugcugcuuua............   7       0    seq
    ...........................................................ccaauauuauugugcugcuuu.............   4       0    seq
    ...........................................................ccaauauuauugugcugcuuua............   2       0    seq
```
